# Seroprevalence of Hepatitis A Virus (HAV) and Hepatitis E Virus (HEV) Among Blood Donor Candidates in Salvador, Bahia, Brazil

**DOI:** 10.3390/v18020162

**Published:** 2026-01-26

**Authors:** Daniela Santana Mendes, Victoria Cruz Paraná, Luan Henrique Paim Santos, Luíza Araújo de Santana Cavalcanti, Júlia Stifelman Freire Alves, Fernanda Souza Novais, Gabriela de Souza Benicio dos Santos, Helen Regina Silva Sodré de Matos, Nelma Pereira Santana, Maria Alice Sant’ Anna Zarife, Carina Carvalho dos Santos, Ricardo David Couto, Maria Isabel Schinoni, André Castro Lyra, Mitermayer Galvão dos Reis, Luciano Kalabric Silva

**Affiliations:** 1 Gonçalo Moniz Institute (IGM), Oswaldo Cruz Foundation (Fiocruz), Salvador 40296-710, Bahia, Brazil; danielamendes.biomedicina@hotmail.com (D.S.M.); victoriaparana@gmail.com (V.C.P.); luan_henrique12@hotmail.com (L.H.P.S.); luiza.ascavalcanti@gmail.com (L.A.d.S.C.); oiju@live.com (J.S.F.A.); fernanda.novaisbio@gmail.com (F.S.N.); gabrielasbs@ufba.br (G.d.S.B.d.S.); mitergreis@gmail.com (M.G.d.R.); 2 Pharmacy Faculty, Federal University of Bahia (UFBA), Salvador 40170-115, Bahia, Brazil; helenssodre@yahoo.com.br (H.R.S.S.d.M.); carvalhos.carina@gmail.com (C.C.d.S.); rdc@ufba.br (R.D.C.); 3 Hematology and Hemotherapy Foundation of the State of Bahia (HEMOBA), Salvador 40286-240, Bahia, Brazil; nelmapsant@uol.com.br; 4 Central Public Health Laboratory of Bahia (LACEN-BA), Salvador 41745-900, Bahia, Brazil; maszarife@gmail.com; 5 Zarns Faculty of Medicine, Medicine FTC, Salvador 41741-590, Bahia, Brazil; 6 School of Medicine, Federal University of Bahia (UFBA), Salvador 40170-115, Bahia, Brazil; mariaschinoni4@gmail.com (M.I.S.); aclyra@live.com (A.C.L.); 7 Department of Epidemiology of Microbial Diseases, School of Public Health, Yale University, New Haven, CT 06511, USA

**Keywords:** hepatitis A (HAV), hepatitis E (HEV), seroprevalence, blood donor candidates, Brazil

## Abstract

Hepatitis A (HAV) and E (HEV) are clinically indistinguishable diseases. This study aimed to determine the seroprevalence of both viruses among 469 blood donor candidates at HEMOBA, Salvador, Brazil. The seroprevalence of anti-HAV IgG was determined by chemiluminescence (Abbott Diagnostics, Chicago, IL, USA) and that of anti-HEV (IgG/IgM) was determined by ELISA (Wantai-Biopharm, Beijing, China). The ineligibility rate was 16.8% (79/469), mostly temporary. There were no statistically significant demographic differences between ineligible and eligible donor candidates. The participants were predominantly male (52.7%), with the skin color Brown/Black (83.2%), and had completed secondary education or started/completed higher education (89.6%). The seroprevalence of anti-HAV IgG was 53.5% (193/361; 95%CI: 48.2–58.7%) among unvaccinated participants; the seroprevalence of anti-HEV IgG was 7.0% (33/469; 95%CI: 4.9–9.7%), and no case of anti-HEV IgM was found. In univariate analysis, HAV was associated with work with livestock and eating fresh fish; HEV was related to low incomes and eating game meat. However, only aging and being born in the interior of the state retained statistical significance in the final model. In conclusion, despite the availability of the HAV vaccine, this study revealed a higher burden of HAV when compared to HEV. Future studies must prioritize risk factor investigations of both viruses in non-metropolitan and rural areas.

## 1. Introduction

Hepatitis consists of an inflammatory process of hepatocytes, which results in varying degrees of liver injury [1]. Its causes include infections by viruses, bacteria, or parasites, with viruses representing the main cause in Brazil’s map of types of hepatitis [2]. Hepatitis viruses (A, B, C, D, and E) share a common tropism for the liver. However, they belong to distinct viral families, possessing genetic and biological structures that do not share a common evolutionary origin. Additionally, they exhibit marked differences in their clinical presentation, epidemiological patterns, and prognosis [3]. They can be classified according to the following: their capacity to cause chronic infections (B, C, and D, and E in immunocompromised patients) or inability to do so (A only); the progression of the disease to cirrhosis and hepatocellular carcinoma (mainly B and C); their primary transmission routes (fecal–oral, A and E; parenteral/infectious, predominantly B, C, and D) [4]. These infections constitute a significant global public health problem, associated with high rates of morbidity and mortality [5].

The hepatitis A virus (HAV) belongs to the *Picornaviridae* family (Genus: *Hepatovirus*) and the hepatitis E virus (HEV) belongs to the *Hepeviridae* family (Genus: *Paslahepevirus*), according to the International Committee on Taxonomy of Viruses (ICTV, [https://ictv.global/], accessed on 12 November 2025). Despite belonging to completely distinct viral families, their clinical presentations are indistinguishable without laboratory confirmation. Although different HAV genotypes (I–III) and HEV genotypes (1–4) can infect humans, this does not translate into significant antigenic diversity, resulting in a single serotype. In general, the host’s immune response generates neutralizing anti-HAV and anti-HEV antibodies, which typically promote spontaneous viral clearance [6]. Consequently, the clinical course for both is generally acute and self-limiting. Most individuals are asymptomatic, and when symptoms do appear, they range from mild to rare, fulminant cases. HEV has been considered a zoonosis, and it has recently been found that the disease can progress to a chronic form, especially in immunocompromised individuals. In addition, the HEV has been associated with extra-hepatic manifestations [7]. Furthermore, HEV can be particularly virulent and severe during pregnancy, with high maternal and fetal morbidity and mortality compared to non-pregnant women. The mortality of HAV cases are approximately the same in pregnant and non-pregnant women [8,9,10].

Infection by HAV and HEV is common in underdeveloped countries with precarious sanitation and hygiene conditions. HEV is hyperendemic in many countries in Asia and Africa and has recently been considered common in developed countries [11,12]. In Brazil, the overall prevalence of HAV can reach 48%, while HEV is less frequent, ranging from 1% to 15% [13,14,15]. In both cases, prevalence tends to increase with age, and its distribution varies according to the geographical area and the study period [13,14,15]. Reports of HAV outbreaks via sexual transmission among adolescents and young adults, particularly among men who have sex with men (MSM), have occurred in recent years in Brazil and globally [16,17]. The WHO estimated that in 2015 and 2016, mortality from HAV and HEV were 0.5% and 3.3% among viral hepatitis patients, respectively [18,19].

Hepatitis B (HBV) and C (HCV), which are transmitted through the parenteral route, are routinely screened in blood donation candidates or in outpatient settings, while HAV and HEV are rarely diagnosed, leading to the underestimation of their prevalence. Although HEV is preferentially transmitted via the enteric route, some studies have shown that HEV can also be transmitted through transfusion (TT-HEV). In one study in southeast England, 1 in every 2848 donors showed viremia at the time of donation [20]. Since 2004, there has been increasing recognition of the risk of TT-HEV in several countries, but its true frequency is underestimated because infected individuals are usually asymptomatic, and the testing of blood donors is absent in the majority of countries [21].

Thus, the present study aimed to investigate the seroprevalence of hepatitis A (HAV) and hepatitis E (HEV) viruses among blood donation candidates at a reference blood transfusion unit in Salvador, Bahia. A differentiated serological approach was strategically defined based on the distinct epidemiological profiles of the two viruses in the region. For HAV, a pathogen with well-established epidemiology in Brazil, the detection of anti-HAV IgG was used to determine previous immunity and exposure. On the other hand, the circulation and true prevalence of HEV are frequently unknown or underestimated in our country. Therefore, the inclusion of anti-HEV IgG and IgM provided a broader assessment, allowing us to simultaneously determine past exposure and obtain an indicator of recent or active viral circulation. Although blood donor candidates do not represent the general population, the evaluation of these serological markers can allow for the estimation of the burden of these diseases and the identification of potential risk factors at the local level.

## 2. Materials and Methods

### 2.1. Study Design

A cross-sectional study was conducted to determine the seroprevalence of anti-HAV IgG and anti-HEV IgG and IgM antibodies in blood donation candidates.

### 2.2. Study Site and Population

This study was carried out at the Hematology and Hemotherapy Foundation of Bahia (HEMOBA), a transfusion reference center located in the HGE Health Complex in Salvador, BA, between 2020 and 2022. After signing informed consent forms, 469 blood donor candidates were recruited to participate in this study. The cohort included participants of both sexes, aged 16 to 67 years, comprising both those eligible and ineligible for donation based on clinical screening. Demographic data and exposure factors were collected through an interview. Blood samples collected during recruitment were submitted to the Molecular Biological Pathology Lab (LPBM) of the Gonçalo Moniz Institute (IGM) at Fiocruz Bahia and to the Laboratory of Clinical and Toxicological Analyses of the Faculty of Pharmacy (LACTFAR/UFBA) for laboratory diagnosis.

Inclusion and exclusion criteria included the following: consent through the signing of the informed consent form (ICF) approved by the CEP/IGM; for illiterate participants, consent was obtained by fingerprint; withdrawal or absence of an interview or the serum sample for testing. Case definitions were established based on serological status. Participants were classified as HAV-prevalent if they presented with anti-HAV IgG reactive samples and had no history of prior vaccination. HEV-prevalent status was assigned to individuals with reactive anti-HEV IgG/IgM, regardless of concurrent ALT and/or AST alteration. Susceptible status was defined by the absence of all relevant serological markers.

### 2.3. Data Collection and Interviews

All participants were interviewed and answered an individual demographic and epidemiological questionnaire. The questionnaire was administered in an appropriate location by trained healthcare professionals. Interview data were registered in a REDCap database (REDCap, http://bdp.bahia.fiocruz.br/, accessed on 12 November 2025) on a dedicated, password-protected computer located at IGM.

### 2.4. Sample Collection and Processing Procedures

Individuals eligible for blood donation had samples collected by venipuncture as part of HEMOBA’s routine transfusion procedure. The serum aliquots from this group were subjected to routine laboratory screening and the detection of anti-HAV IgG and anti-HEV IgG/IgM antibodies. In contrast, for candidates deemed ineligible for donation, collection was performed separately, after clinical dispatch, using vacuum tubes. All sample collections were performed by trained HEMOBA professionals.

### 2.5. Serological Diagnosis

The detection of anti-HAV IgG antibodies was evaluated using the automated chemiluminescence microparticle immunoassay (CMIA) on the Architect i2000SR system (Abbott Diagnostics, Chicago, IL, USA), according to the manufacturer’s instructions. Using this method, microparticles coated with HAV antigen capture IgG antibodies present in the sample, which are detected by an anti-human conjugate supplemented with a chemiluminescent marker. The resulting chemiluminescent reaction is measured as relative light units (RLUs). Samples were analyzed based on information from the manufacturer: RLU < 1.00 nonreactive; RLU ≥ 1.00 reactive. According to information from the manufacturer, the test presented a specificity of ≥99.17% and a sensitivity of ≥98%.

Furthermore, the presence of anti-HEV IgG and IgM antibodies was confirmed by indirect and capture ELISA, respectively, according to the manufacturer’s instructions (Wantai-Biopharm, Beijing, China). To detect anti-HEV IgG, plates pre-coated with HEV recombinant antigens were incubated with serum samples and controls. During the first incubation step performed at 37 °C for 30 min, anti-HEV IgG-specific antibodies, if present, should bond to the antigens. The wells were washed to remove unbound serum proteins, and then rabbit anti-human IgG antibodies (anti-IgG) conjugated to horseradish peroxidase (HRP-conjugate) were added. During the second incubation step performed at 37 °C for 30 min, these HRP-conjugated antibodies bound to any previously formed antigen–antibody (IgG) complexes, and the unbound HRP-conjugate is then removed by washing. Chromogen solutions containing urea peroxide (solution A) and Tetramethylbenzidine (TMB) (solution B) were added to the wells. During the third and final incubation step performed at 37 °C for 15 min, in presence of the antigen–antibody-anti-IgG (HRP) immunocomplex, the colorless chromogens were hydrolyzed by the bound HRP conjugate to a blue-colored product. The blue color turned yellow after stopping the reaction with sulfuric acid. Wells containing negative specimens for anti-HEV IgG remained colorless. Each incubation step was carried out in a microplate incubator (NI 1720, Nova, Piracicaba, Brazil), the washing steps were conducted on an RT3000 washer (Rayto, Shenzhen, China), and the optical density was determined at 450 nm on the RT6000 spectrophotometer (Rayto, Shenzhen, China).

To detect anti-HEV IgM, plates pre-coated with antibodies directed to human immunoglobulin M proteins (anti-μ chain) were first incubated with serum samples and controls, where any IgM-class antibodies were captured in the wells. After washing out all the other substances of the specimen, and, in particular, IgG-class antibodies, the specific HEV IgM captured on the solid phase was detected by the addition of recombinant HEV ORF2 antigen conjugated to the enzyme horseradish peroxidase (HRP-conjugate). In the presence of the (anti-μ)–(HEV-IgM)–(HEV Ag-HRP) immunocomplex, the colorless chromogens were hydrolyzed by the bound HRP-conjugate to a blue-colored product. The blue color turned yellow after stopping the reaction with sulfuric acid. Wells containing negative specimens for anti-HEV IgM remained colorless. All incubation, washing, and coloring steps were performed as described above using the same apparatus. In the indirect and capture ELISAs, the amount of color intensity could be measured and was proportional to the amount of the anti-HEV IgG specific antibodies or the anti-HEV IgM antibody captured in the wells, respectively. Specimens with (A) values less than the cut-off (CO) value of each kit are negative for these assays (A/CO < 1). Specimens with (A) values equal to or greater than the CO value are considered initially reactive (A/CO ≥ 1). Specimens with an A value-to-CO ratio between 0.9 and 1.1 were considered borderline and the retesting of these specimens in duplicate was performed to confirm the initial results. According to information from the manufacturer, the Wantai anti-HEV IgG presented a specificity of 99.9% and a sensitivity of 100%, while the anti-HEV IgM presented a specificity range of 95.3~100.0% in 11 non-hepatitis E groups (included hepatitis A cases, hepatitis B cases, hepatitis C cases, groups inoculated with the HBV vaccine, populations with routine virus testing, blood donors and healthy individuals) and a sensitivity of 97.1%.

### 2.6. Biochemical Dosages

The activities of alanine aminotransferase (ALT) and aspartate aminotransferase (AST) were determined in serum samples using commercial colorimetric assay kits: the Alanine Transaminase Activity Assay Kit (ab105134, Abcam, Cambridge, UK) and the Aspartate Transaminase Activity Assay Kit (ab105135, Abcam, Cambridge, UK), respectively. All assays were performed strictly according to the manufacturer’s instructions. The results were expressed in international units per liter (IU/L).

### 2.7. Data Analysis

Statistical analysis was conducted using Epi Info 7.2.7.0 software (CDC, Atlanta, GA, USA). The events of interest were described using frequencies and measures of central tendency (mean, median) and dispersion (standard deviation). The 95% Confidence Interval (95% CI) with continuity correction was calculated using the VassarStats website (Proportions → The Confidence Interval of a Proportion), available at http://vassarstats.net/ accessed on 12 November 2025. Chi–square for trend was used to test the association between the seroprevalence and age group. For the univariate analysis of categorical variables, the Chi-squared test (with Yates’s correction) or Fisher’s exact test was employed when applicable. For continuous variables, the non-parametric Kruskal–Wallis test was utilized. The strength of association between seropositivity (dependent variable) and the independent variables was quantified by calculating the crude *p*-value. Variables significantly associated in the univariate analysis were included in a multivariate logistic regression model to estimate the adjusted *p*-value. A significance level of 5% (*p* < 0.05) was used for all statistical tests.

## 3. Results

**Figure 1:**
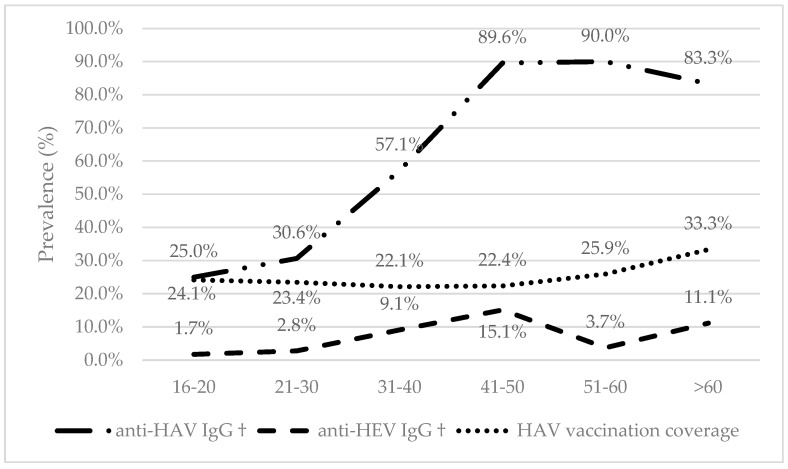
Distribution of anti-HAV (unvaccinated) and anti-HEV IgG positivity, and HAV vaccination coverage, among blood donor candidates by age group, HEMOBA, Salvador, Brazil, from 2020 to 2022 (n = 468 *). * Age was unknown in one participant. † Chi–square for trend *p* < 0.01.

A total of 469 blood donor candidates participated in this study and were tested for HAV and HEV (Table 1). Key sociodemographic and laboratory characteristics are described in Table 1. The donor ineligibility rate was 16.8% (79/469), primarily due to temporary conditions (tattoos, piercings, or micropigmentation less than 1 year old, antibiotic treatment, etc.). There were no statistically significant demographic differences between ineligible and eligible donor candidates. The participants were predominantly male (52.7%), with a mean age of 33.4 years (standard deviation, SD ± 11.1 years of age), ranging from a minimum of 16 to a maximum of 67 years. Regarding skin color/race, the majority self-identified as Brown/Black (83.2%), White (15.1%), and Yellow or Amerindian (1.5%). Most participants reported 12 years or more of schooling, corresponding to having completed secondary education and having started or completed higher education (89.6%). Regarding the anti-HAV IgG status, it is not a criterion for blood donation ineligibility. However, this serological marker cannot distinguish between natural exposition (n = 361) and vaccine-induced immunity (n = 108). The HAV mean vaccination coverage was 23.0% (108/469) and was similar across the decennial age groups (Figure 1). There was no significant difference in the mean age (34.3 ± SD 11.2 vs. 32.7 ± SD 10.8) or proportion of male participants (43.7% vs. 56.3%) between the group with no or unknown vaccination history against HAV (unvaccinated). Therefore, to provide the most accurate estimate of true past infection, the HAV prevalence was calculated solely using the natural exposure group. The seroprevalence of anti-HAV IgG was estimated at 53.5% (193/361; 95% CI:48.2–58.7%) among the unvaccinated individuals. The seroprevalence of anti-HEV IgG in the overall population was much lower at 7.0% (33/469; 95% CI: 4.9–9.7%). Although some participants presented altered aminotransaminase serum levels, there were no cases of anti-HEV IgM reactivity.

Although HAV and HEV are classically transmitted via the fecal–oral route, both can also be disseminated through parenteral exposure. Few participants reported having received a blood transfusion, noting that HAV and HEV are not routinely screened for in blood banks. None presented with chronic kidney disease or a history of organ transplantation. However, potential percutaneous risk factors were observed, such as prior use of non-disposable syringes (5.3%), the presence of piercings (17.9%), tattoos (36.5%), use of surgeries (46.1%), and invasive dental procedures (56.8%).

Lifestyle habits and environmental exposures were also investigated. Most participants reported consuming alcoholic beverages (64.5%) and having an active sexual life (95.1%), with irregular condom use (80.5%). The use of injectable (0.2%) or inhaled drugs (6.2%) was virtually absent. All participants resided in households with water supply and sewage systems, and few reported using water from wells or rivers (2.3%). Despite predominantly living in urban areas, some participants reported having faced floods (24.0%) and direct contact with livestock (17.5%), suggesting possible environmental and occupational sources of HEV exposure.

The seroprevalence of HAV increased progressively with age, peaking in the fifth and sixth decades of life, between 41 and 50 years (89.6%) and between 51 and 60 years (90.0%). There was a slight reduction in the age group over 60 years (83.3%). Exposure to HEV also accumulated with age, with rates gradually increasing until reaching a peak in the fifth decade of life, between 41 and 50 years (15.1%), but then significantly decreased in the sixth decade (3.7%) and rose again among those older than 60 years (11.1%) (Figure 1).

**Figure 2:**
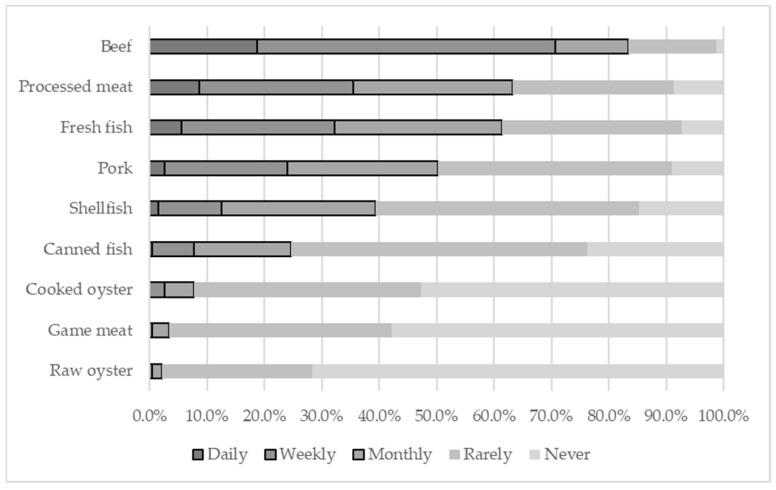
The distribution of the relative frequency of consumption of foods from different origins among blood donor candidates at HEMOBA, Salvador, Brazil, from 2020 to 2022 (n = 469).

Given the established food-borne and waterborne transmission routes of both viruses, the relative frequency of consumption of foods from different origins reported by the candidates was dichotomized into two categories: ‘Frequent’ (combining responses of daily, weekly, and monthly consumption) and ‘Infrequent’ (combining rare and no consumption). Among the most frequently consumed foods were beef (83.3%), processed meat/sausages (63.0%), and fresh fish (61.1%). A smaller number of participants also reported including pork (50.2%), shellfish/seafood (39.0%), and canned fish (24.5%) and an even smaller number reported consuming cooked oysters (7.8%), game meat (3.4%), and raw oysters (2.1%) in their diet (Figure 2).

In univariate analysis, both viruses were associated with higher age (Figure 1). Seroprevalence to anti-HAV IgG was also associated with being born in the interior of the state (67.5% vs. 46.5%; crude *p*-value < 0.01) and independently associated with having worked with livestock (74.6% vs. 48.5%; crude *p*-value = 0.04) and frequent consumption of fresh fish (74.6% vs. 48.5%; crude *p*-value = 0.04) (Table 2). Seroprevalence to anti-HEV IgG was also associated with being born in the interior of the state (11.8% vs. 4.7%; crude *p*-value < 0.01); however, it was independently associated with a monthly family income less than or equal to three Brazilian minimum wages (SM) (9.1% vs. 4.1%; crude *p*-value = 0.04) and frequent consumption of game meat (25.0% vs. 6.2%; crude *p*-value = 0.02) (Table 2). Sociodemographic variables (sex, skin color, and education level) (Table 2), along with factors such as blood contamination, lifestyle habits, and environmental exposures, were also evaluated. None of these variables demonstrated an association with either virus (*p* > 0.05). In the multivariate logistic regression analysis, only aging and being born in the interior of the state retained statistical significance in the final model (adjusted *p*-value < 0.05) (Table 2).

## 4. Discussion

Studies involving blood donor candidates are commonly used as a convenience population-based sample, allowing estimates of the burden of various diseases [14,22]. However, this population is known to be biased, predominantly composed of healthy individuals, with a minimum age of 16 years and a maximum of 69 years, which limits analyses at the extremes of age. In a population-based study conducted in Florianópolis, 30% of participants reported having donated blood at some point in their lives, while only 6% had donated in the previous 12 months [23]. Despite these limitations, prevalence estimates obtained among donors can often be extrapolated to the general population. For instance, the seroprevalence of hepatitis C in Salvador among blood donors in 1995 was 1.7% [24] and in the general population in 2006 it was 1.5% [25].

No significant differences were observed between eligible and ineligible donor candidates, except for a predominance of females among the ineligible group. This pattern contrasts with findings from a study conducted in Rio Grande do Sul, which reported a higher proportion of ineligible male candidates associated with multiple sexual partners and high-risk behaviors [15]. The sex difference observed in our study may reflect a selection bias, potentially stemming from male participants prematurely withdrawing from the comprehensive blood collection and interview process more frequently than females, leading to the loss of these potential participants before final documentation. Conversely, a major contributor to female ineligibility was the frequent observation of low hematocrit, largely attributable to the menstrual cycle [26,27]. Importantly, the lack of association between HAV and HEV exposure and sex suggests that neither this selection bias nor the physiological factors (hematocrit) substantially affected the primary outcome regarding viral exposure.

Blood and organ donation is frequently considered an altruistic act, crucial for saving lives in emergencies, complex surgeries, transplants, or treating individuals with chronic diseases. The participants had a demographic profile reflective of Salvador’s general population, being predominantly of Brown/Black skin color (African descent). Salvador is widely known as “Brazil’s Blackest city”. However, a series of inequities in Brazil are often related to the socioeconomic status and the race/skin color of individuals served by the Brazilian public health system (SUS) [28]. In contrast, the cohort exhibited significantly higher levels of education, suggesting that this elevated education attainment may be associated with the altruism of blood donation. A similar finding was reported in other studies, which emphasize the role of cultural and personal factors in influencing the decision to donate blood and organs [29].

Concerning serology, anti-HAV IgG was detected in 55.2% of participants, although some cases may have acquired immunity through vaccination (self-reported). Since 2014, Brazil has implemented HAV immunization in two-year-old children, with a high seroconversion rate, making post-vaccination testing generally unnecessary. Self-reported vaccine coverage in our sample was lower than that observed in children (>65% [30] vs. a mean of 23.0% in this study), likely due to vaccine unavailability for adolescents and adults at the time of this study. The seroprevalence of anti-HAV IgG was estimated at 53.5% among the unvaccinated individuals. The prevalence of hepatitis A has decreased in Brazil in recent decades, but children and adolescents from lower socioeconomic classes are still more susceptible; it is a disease that tends to be asymptomatic in children, but adolescents and adults present symptoms [31]. The overall prevalence of HAV can reach 85.9%; it is lower in children and adolescents than in adults and higher in rural areas compared to urban areas. Its distribution variation according to geographic area and study period is between 30.8% and 85.9% [15,32,33]. The seroprevalence by age group was similar to those of previous studies in Brazil, where exposure to HAV increases with age [32]. Critically, this cohort reflects a significant epidemiological shift: Historical data reported seroprevalence rates above 90% among young adults around 20 years of age. In our cohort, only approximately 25% of individuals in this age group were immune, leaving nearly 75% susceptible. Although overall HAV-related mortality has decreased over recent decades, susceptible adults are at increased risk of severe disease. The case fatality rate (CFR) for HAV is typically low (around 0.3% to 0.8% of symptomatic cases); however, it significantly increases with advancing age, reaching up to 2.2% in individuals over 50 years old. This dramatic shift in susceptibility, coupled with the age-dependent increase in CFR, strongly reinforces the need for targeted vaccination strategies and continued surveillance in susceptible adult populations [3,34,35].

The seroprevalence of anti-HEV IgG was around 7.0%, with no acute cases detected by anti-HEV IgM. This finding places our study’s prevalence within the range reported in other previous Brazilian investigations. HEV seroprevalence rates among blood donors in the country reveal sharp regional contrasts, such as the variations observed between Pernambuco (0.9%), Paraná (2.3%), Mato Grosso do Sul (6.4%), São Paulo (9.8%), and Santa Catarina (10.0%) [36,37]. In the past, comparisons across studies could be limited by the variable performance of anti-HEV IgG detection assays [38]. However, with the progressive improvement of the assays, we now believe that the significant geographical heterogeneity of HEV exposure across Brazil primarily reflects true regional differences in transmission routes (e.g., sanitation access, animal exposure). European studies indicate an increase in HEV seroprevalence with age, possibly influenced by factors such as the type of serological test and dietary habits [39]. In our study, the seroprevalence of HEV also increased with age, peaking in the fourth decade, but it declined in the fifth decade, before rising again in the sixth decade. One could speculate that this is due to the long-term persistence of antibodies, the potential influence of immunosenescence, and a growing susceptibility to infection at older ages. If this is the case, much attention should be paid to older susceptible individuals, especially those immunosuppressed individuals with HIV and hematological malignancy and transplant recipients. The disease usually presents as a self-limiting acute illness, but recently chronic HEV infection has been increasingly reported in immunocompromised hosts, especially after solid organ transplantation [40]. Hepatic markers (ALT and AST) remained normal among most participants (>95%). Although sensitive to liver injury, these enzymes are not specific for viral hepatitis and may increase during prodromal phases without direct relation to disease severity. Despite the risk being considered low, several countries have implemented universal or selective HEV-RNA screening to prevent the risk of TT-HEV [41].

Our study identified widespread circulation of both viruses in the population of blood donor candidates. While no clear association was observed between HAV and HEV exposure, their distinct epidemiological profiles suggest different risk factors that warrant further investigation. Both viruses are known to share the fecal–oral route as their primary means of transmission, although HEV is considered a zoonosis and is implicated in vertical transmission with consequences for both mother and baby [10]. The reduction in the burden of HAV in Brazil has been associated with improvements in sanitary conditions and the introduction of the HAV vaccine in the national immunization program [3,34]. Conversely, HEV represents a significant contemporary concern, primarily due to its infrequent diagnosis, the lack of a vaccine, and the absence of screening in blood banks. Although the recombinant HEV vaccine (Hecolin, also designated HEV 239 vaccine) is licensed in China, it is not available in Brazil. Regarding diet, studies in developed countries show that HAV occurs mainly through food and water contamination, whereas HEV is frequently associated with pigs or wild animals [42]. Among the findings of this study, frequent consumption of fresh fish was associated with HAV exposure, while HEV exposure was exclusively linked to the consumption of game meat. Furthermore, critical questions regarding hygiene practices, food preparation methods, and the specific role of pork consumption warrant further investigation within a ‘One Health’ context.

## 5. Conclusions

Despite the availability of a vaccine, HAV showed a higher population burden than HEV. Exposure to both viruses increased with age and was associated with common socio-environmental factors and specific habits, including rural residence and contact with animals. In the case of HAV, it may indirectly reflect hygiene issues since HAV is not a zoonotic disease. On the other hand, HEV was not associated with swine contact or the consumption of pork meat in our setting, but it was associated with consuming game meat. Future studies must prioritize risk factor investigations of both viruses in non-metropolitan and rural areas.

## Figures and Tables

**Table 1:** Demographic and laboratory characteristics of blood donor candidates, HEMOBA, Salvador, Brazil, from 2020 to 2022 (n = 469).

Characteristics	n = 469 ^1^
Donor candidates	
Ineligible	79 (16.8)
Eligible	390 (83.2)
Sex	
Male	247 (52.7)
Female	222 (47.3)
Age (years), mean	33.4 (11.1)
Range	16–67
Skin color	
Brown	212 (45.2)
Black	178 (38.0)
White	71 (15.1)
Yellow	5 (1.1)
Indigenous	2 (0.4)
Education level	
<12 years	49 (10.4)
≥12 years	420 (89.6)
History of HAV vaccination	
Yes	108 (23.0)
No ^2^	144 (30.7)
Unknown ^2^	217 (46.3)
Seroprevalence	
Anti-HAV IgG ^2^	193 (53.5)
Anti-HEV IgG	33 (7.0)
Anti-HEV IgM	0 (0.0)

^1^ n (%); Mean (SD). ^2^ Unvaccinated was defined as having no or unknown vaccination history against HAV (n = 361).

**Table 2:** Exposure factors associated with the seroprevalence of anti-HAV and anti-HEV IgG among blood donor candidates at HEMOBA, Salvador, Brazil, from 2020 to 2022 (n = 469).

Characteristics	Anti-HAV IgG			Anti-HEV IgG		
	Prevalence (%) ^1^	Crude *p*-Value	Adjusted *p*-Value	Prevalence (%) ^2^	Crude *p*-Value	Adjusted *p*-Value
Overall	193/361 (53.5)			33/469 (7.0)		
Sex						
Male	112/215 (52.1)	0.59	-	21/247 (8.5)	0.21	-
Female	81/146 (55.5)			12/222 (5.4)		
Born						
Interior	81/120 (67.5)	<0.01	0.02	18/153 (11.8)	<0.01	<0.01
Salvador	112/241 (46.5)			15/316 (4.7)		
Monthly family income						
<1–3 SMs	112/201 (55.7)	0.33	-	24/265 (9.1)	0.04	0.93
>3 SMs	77/154 (50.0)			8/196 (4.1)		
Skin color						
Brown/black	164/301 (54.5)	0.39	-	30/390 (7.7)	0.33	-
White	28/59 (47.5)			3/78 (3.8)		
School years						
<12 years	19/39 (48.7)	0.61	-	3/49 (6.1)	1.00	-
≥12 years	174/322 (54.0)			30/420 (7.1)		
Worked with livestock						
Yes	50/67 (74.6)	<0.01	0.04	8/82 (9.8)	0.34	-
No	142/293 (48.5)			25/386 (6.5)		
Eating fresh fish						
Frequently	129/224 (57.6)	0.04	0.16	18/287 (6.3)	0.52	-
Rarely/never	63/136 (46.3)			15/181 (8.3)		
Eating pork meat						
Frequently	92/183 (50.3)	0.25	-	16/235 (6.8)	0.98	-
Rarely/never	100/177 (56.5)			17/233 (7.3)		
Eating game meat						
Frequently	8/12 (66.7)	0.40	-	4/16 (25.0)	0.02	0.26
Rarely/never	183/347 (52.7)			28/451 (6.2)		

^1,2^ The total varies depending on the availability of data. ^1^ Unvaccinated was defined to have no or unknown vaccination history against HAV (n = 361). SM = Brazilian minimum month wage is BRL 1518.00, which corresponds to approximately USD 283.00.

## Data Availability

The datasets generated and analyzed during the current study are available from the corresponding author on reasonable request. Due to privacy and ethical restrictions, individual-level data cannot be publicly shared.
